# Biodistribution and function of coupled polymer-DNA origami nanostructures

**DOI:** 10.1038/s41598-023-46351-1

**Published:** 2023-11-10

**Authors:** Noah Joseph, Anastasia Shapiro, Ella Gillis, Shirin Barkey, Almogit Abu-Horowitz, Ido Bachelet, Boaz Mizrahi

**Affiliations:** 1 Augmanity Nano Ltd., 7670308 Rehovot, Israel; 2 Faculty of Biotechnology and Food Engineering, 32000 Technion, Haifa Israel

**Keywords:** Biotechnology, Structural biology

## Abstract

Spatial control over the distribution of therapeutics is a highly desired feature, which could limit the side effects of many drugs. Here we describe a nanoscale agent, fabricated from a coupled polymer-DNA origami hybrid that exhibits stability in serum and slow diffusion through tissues, in a manner correlating with shape and aspect ratio. Coupling to fragments of polyethylene glycol (PEG) through polyamine electrostatic interactions resulted in marked stability of the agents in-vivo, with > 90% of the agents maintaining structural integrity 5 days following subcutaneous injection. An agent functionalized with aptamers specific for human tumor necrosis factor TNF-alpha, significantly abrogated the inflammatory response in a delayed-type hypersensitivity model in humanized TNF-alpha mice. These findings highlight polymer-DNA hybrid nanostructures as a programmable and pharmacologically viable update to mainstream technologies such as monoclonal antibodies, capable of exerting an additional layer of control across the spatial dimension of drug activity.

## Introduction

Many drugs, including small molecules and biologicals, operate systemically with no innate control over distribution and, therefore, function. This lack of control is a central driver of adverse effects, and a major component in failures of many new drugs in clinical trials^1^, as well as in decisions to withdraw drugs that entered clinical use^2^. Although great efforts have been made in the past decades to achieve layers of control over drug activity, the current arsenal of approved drugs represents a small fraction of the true potential of drug control mechanisms. It certainly does not achieve the level of arbitrary control exhibited by technologies such as microelectromechanical systems (MEMS) or logic circuits in computers.

Monoclonal antibodies (mAbs), a mainstream and well-proven pharmaceutical technology, exemplify this challenge well. Monoclonal antibodies enabled breakthrough treatments in diseases that have until then been considered nearly untreatable. Since the 1980s, mAbs are increasingly being incorporated into clinical practice as therapeutic options, particularly in oncology, immunology and inflammatory diseases^3,4^. Many technical efforts, including molecular engineering, proteomics and genomics approaches have been made to generate and optimize therapeutic antibodies and antibody-based fusion molecules with decreased immunogenicity and with improved efficacy of effector functions^5,6^. However, they are to date still administered systemically, which has led to disqualifying adverse reactions in major clinical trials such as the appearance of tuberculosis or lymphomas, the worsening of heart failure, and increased cardiocytotoxicity^7–10^. Antibodies disperse rapidly and only a low percentage of patients exhibit long-lasting complete response to treatments, particularly in cancer^11–14^. A solution for spatially confining and controlling their behavior, leading to focusing and enhancing the desired effect, would be highly desirable.

One such solution, which is particularly suitable for monoclonal antibodies, could be coupling to biocompatible, stable agents with programmable spatial distribution. The resulting conjugate maintains antibody activity, i.e. binding and neutralization of soluble or membrane-bound targets, while restricting the diffusion of the antibody away from its target site or tissue, increasing its concentration there and limiting its occurrence elsewhere.

Scaffolded DNA origami is a method for fabrication of DNA nanostructures enabling precise spatial control, programmability and functionality at sub-nanometer resolution^15,16^. These unique properties make them popular in various research fields, and mark them as the next generation therapeutic and diagnostic agents^15–22^. Briefly, in this technique a long single stranded DNA scaffold is folded by position-specific hybridizations of short oligonucleotides, which serve as staples into a predesigned structure^23^. Different active moieties (i.e., aptamers, proteins, DNAzymes, etc.) can be attached easily and simultaneously on the nanostructures at desired sites and orientation, allowing different levels of control and functionality. Mass production of DNA origami in a cost-efficient manner has already been demonstrated^24^, whereas pharmacological concerns/challenges can be addressed by coating the structures with protective polymers such as Polyethylene glycol (PEG)^25–28^.

Specific examples of applications of such constructs could be envisioned. One example is the functionalization of DNA origami nanostructure with multiple copies of protein-neutralizing aptamers. The size of some typical DNA origami nanostructures (on the order of several 10 to several 100 nm) enables the attachment of multiple aptamer types, which could achieve higher functional complexity compared with monoclonal antibodies. Indeed, the usage of mAb cocktail is also possible; however, the actual cost of development and production of such therapeutic mAb combination may render this approach unrealistic, while DNA origami-aptamer based agents are modular in nature.

Here we present a strategy to confer spatial control over drug activity, based on coupled polymer-DNA origami hybrid nanoscale agents. The size, shape, and aspect ratio of these agents, which are completely designed and range from 14 × 27 × 61 to 8.1 × 11.7 × 215.2 nm dictate specific diffusion rates through tissues. We use fragments of PEG-polylysine to coat the DNA origami structures, creating a PEG-DNA hybrid for enhanced in-vivo stability of the agents. We functionalized the agents by folding specific aptamers (either DNA- or RNA-based) into the origami fabrication process. Following visual, kinetic, and stability characterization of several DNA origami agents in-vivo, an optimal DNA nanostructure was selected as a proof of principle towards therapeutic application and presented a highly-potent anti-inflammatory effect in a humanized TNFalpha (TNFa) mouse model of TNCB-induced delayed-type hypersensitivity reaction (DTHR) by targeting human TNFa (hTNFa). The design reported here can be adapted rapidly to target other proteins which are involved in a variety of diverse pathologies, and could lead to a new type of programmable agents for a wide range of therapeutic applications.

## Results

**Figure 1:**
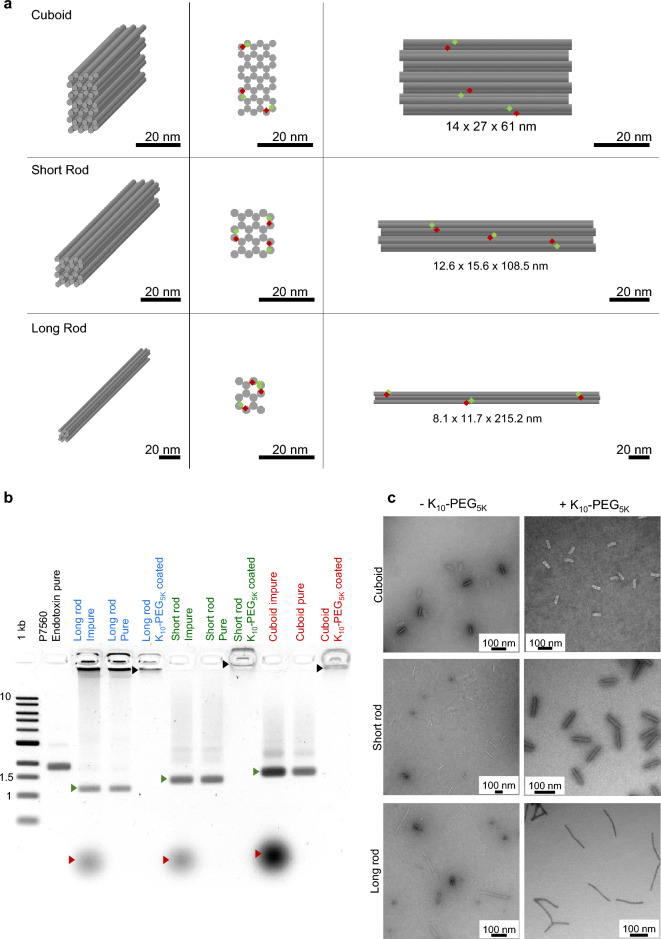
Structural characterization of the DNA origami nanostructures and quality assessment of their assembly. (**a**) Design schematic. The rows show the different DNA origami nanostructures investigated: cuboid, short rod and long rod (from top to bottom). The columns show different views of the DNA origami nanostructures: 3D, front and side view (from left to right). FRET pairs are distributed evenly on the DNA origami nanostructures and shown as red (Atto 647N) and green (Atto 488) diamonds. All scale bars are 20 nm. (**b**) Quality evaluation of the DNA origami nanostructures after assembly (lanes 3, 6, 9), after PEG purification (lanes 4, 7, 10) and after PEG-polylysine addition (lanes 5, 8, 11) as analyzed by gel electrophoresis. 1 kb double-stranded DNA was used as a ladder and specific bands are indicated, numbers are in kb. Scaff. P7560 ssDNA scaffold. Red arrows indicate staple excess and leftovers, green arrows represent the well-folded nanostructures before and after PEG purification, and the black arrows show the purified nanostructures coated with PEG_5K_-K_10_. (**c**) DNA origami nanostructures as visualized by transmission electron microscopy (TEM). Each structure was imaged before and after PEG-Poly(lysine) coating as indicated. All scale bars are 100 nm.

As a starting point for this proof-of-feasibility study, we chose 3 different structures of the same mass (~ 4.9 MDa), which represent a range of aspect ratios in symmetric shapes with simple geometry: a cuboid (14 × 27 × 61 nm,), a short rod (12.6 × 15.6 × 108.5 nm) and a long rod (8.1 × 11.7 × 215.2 nm). Each shape was designed with staples harboring 3 FRET pairs (based on Atto 488 as donor and Atto 647N as acceptor), and was assembled, PEG purified and coated with PEG-polylysine to render it stable towards various solutions, followed by endotoxins removal (Fig. 1a, Supplementary Fig. S1, Supplementary Table S1). Samples were analyzed using agarose gel electrophoresis to determine the bulk quality following each step, i.e.; after assembly, after PEG purification and after PEG-polylysine addition (Fig. 1b, Supplementary Fig. S2). As can be seen, following assembly, each of the folded DNA nanostructures showed a strong leading band with an excess of staple strands being visible at the bottom of the gel. After PEG purification, a clear leading band of the desired structure is visible at the same height of the band detected directly after assembly, with staples being successfully removed by PEG purification. Coating each of the DNA nanostructures using PEG-polylysine (PEG_5K_-K_10_) through amine (positively charged Lysine) and phosphate (negatively charged DNA backbone) interactions resulted in increased mass and absence of migration within the agarose gel, proving the successful attachment of PEG and stabilization of the DNA origami nanostructure (Fig. 1b, Supplementary Fig. S2). Proper folding and structural integrity was verified and determined by transmission electron microscopy (TEM) before and after PEG-polylysine coating (Fig. 1c).

**Figure 2:**
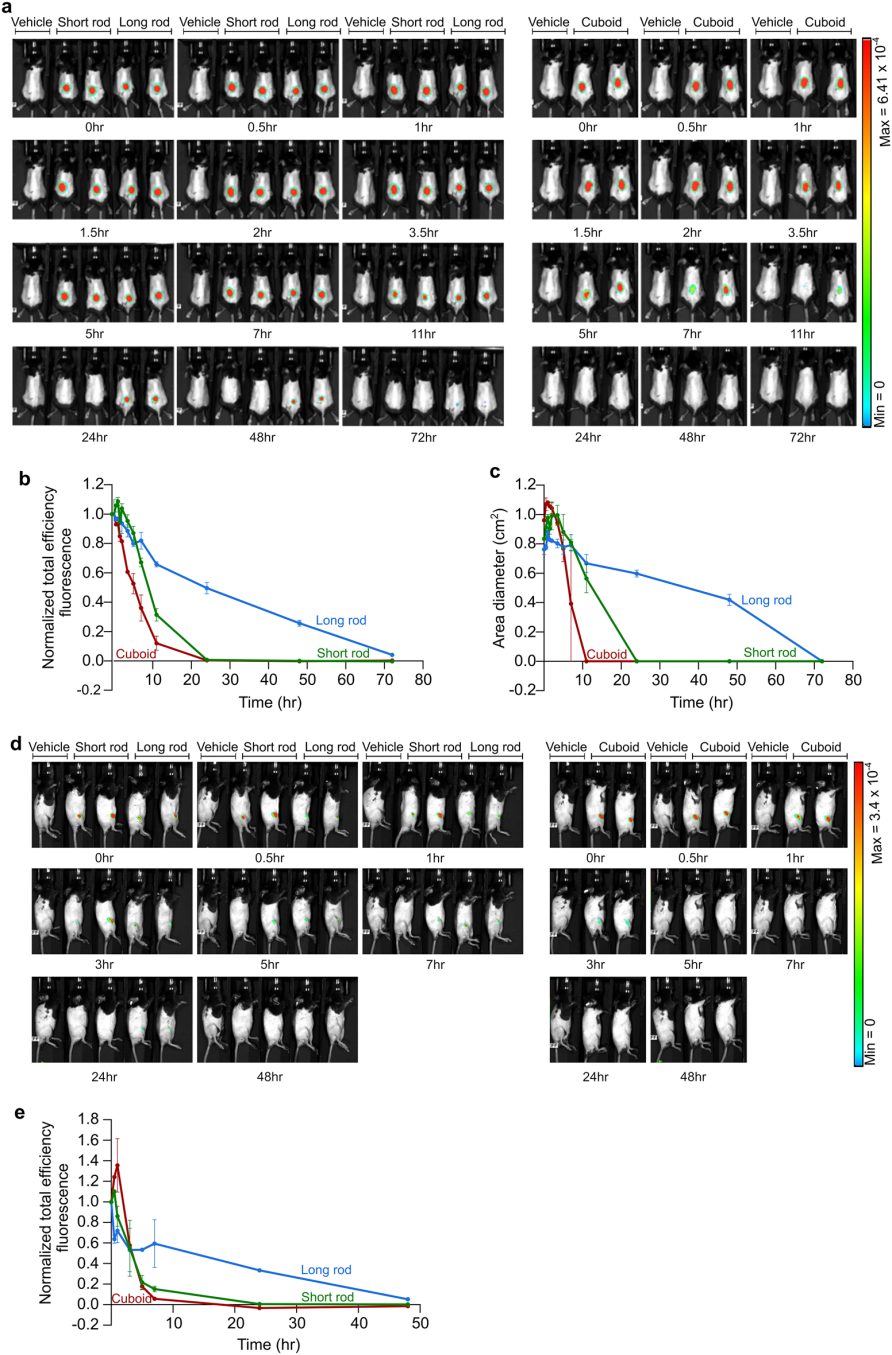
Biodistribution of different DNA origami nanostructures. (**a**) Live image analysis of total body biodistribution over time of the indicated DNA origami nanostructures following their subcutaneous injection into mice. Heat map false color correlates to FRET levels. (**b**) Quantification of total efficiency fluorescence obtained in mouse images from A. Same region of interest (ROI) was chosen around the injection area for each mouse and the FRET fluorescent total efficiency of the indicated DNA origami nanostructures was measured in each ROI along time points. Calculations were performed as described in “Methods”. Data presented are the mean values ± SEM. (**c**) Quantification of the indicated DNA origami nanostructure diffusion along time following their subcutaneous injection into mice. Calculations were performed as described in “Methods” based on mouse images from A. Data presented are the mean values ± SEM. (**d**) Live image analysis of total body biodistribution over time of the indicated DNA origami nanostructures following their injection into mouse knee joints. Heat map false color correlates to FRET levels. (**e**) Quantification of total efficiency fluorescence obtained in mouse images from D. Same region of interest (ROI) was chosen around the injection area for each mouse and the FRET fluorescent total efficiency of the indicated DNA origami nanostructures was measured in each ROI along time points. Calculations were performed as described in “Methods”. Data presented are the mean values ± SEM.

A key aspect of the applicability of any drug is its in-vivo stability and distribution. We therefore performed live imaging of mice treated with either the PEG-polylysine-coated cuboid, short rod, or long rod nanostructures administered either subcutaneously, into the knee joints, or intraperitoneally into mice. Image intensity was quantified over 3 days, and showed higher stability of the long rod compared with the cuboid and short rod nanostructures when injected subcutaneously or into the knee joints (Fig. 2, Supplementary Fig. S3a). Moreover, the long rod demonstrated an extended diffusion over time, indicating slower diffusion combined with greater stability when administered subcutaneously (Fig. 2c).

**Figure 3:**
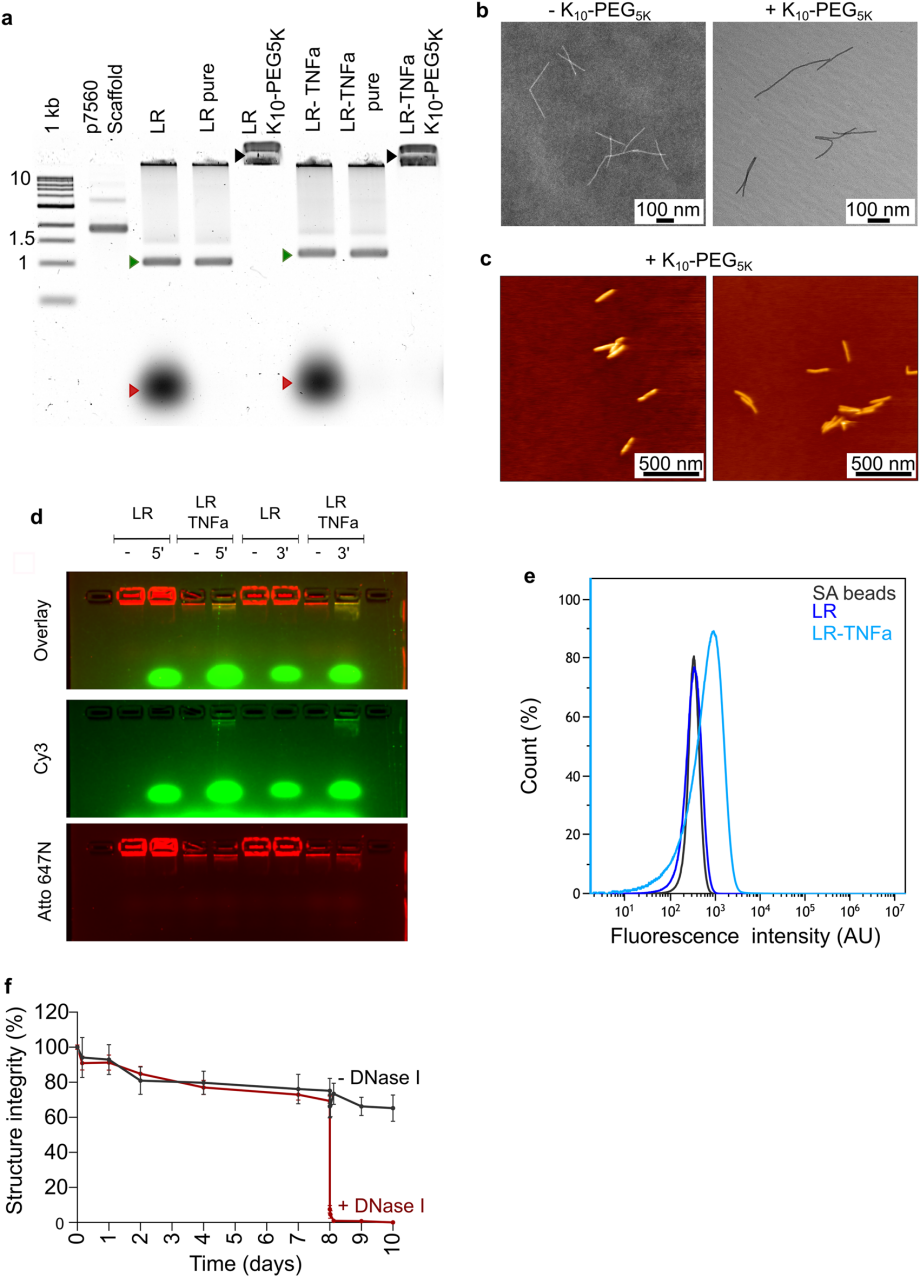
Characterization of the long rod-TNFa aptamer structure, specificity and stability. (**a**) Quality evaluation of either the long rod-no aptamer (LR) or the long rod-TNFa aptamer (LR-TNFa) after assembly (lanes 3, 6), after PEG purification (lanes 4, 7) and after PEG-polylysine addition (lanes 5, 8) as analyzed by gel electrophoresis. 1 kb double-stranded DNA was used as a ladder and specific bands are indicated on the left side, numbers are in kb. Scaff., P7560 ssDNA scaffold. Red arrows indicate staple excess and leftovers, green arrows represent the well-folded nanostructures before and after PEG purification, and the black arrows show the purified nanostructures coated with PEG_5K_-K_10_. (**b**) TEM image of the long rod-TNFa aptamer before and after PEG-Poly(lysine) coating as indicated. All scale bars are 100 nm. (**c**) Representative AFM images of the long rod-TNFa aptamer. Height is coded by color. All scale bars are 500 nm. (**d**) Incubation of a reverse complement sequence for the TNFa aptamer with either the long rod-no aptamer (LR) or the long rod-TNFa aptamer (LR TNFa) followed by gel electrophoresis. 5ʹ or 3ʹ indicates the end at which the reverse complement oligonucleotide was tagged with Cy3. (**e**) FACS analysis of long rod binding to recombinant human TNFa protein-coated streptavidin (SA) beads. (**f**) Time-dependent stability of the long rod-TNFa aptamer in human serum. Half of the samples were treated with DNase I 192 h following incubation. Structure integrity was calculated as described in “Methods”. Data presented are the mean values ± SEM.

Based on the kinetics and the in-vivo stability findings, we selected the PEG-polylysine-coated long rod nanostructure for further efficacy and druggability experiments. The long rod was slightly redesigned to comprise 20 copies of a human TNFa (hTNFa) aptamer, which were anchored and distributed uniformly across the structure’s surface (Supplementary Table S2). Similar to all other origami structures, this version was assembled, PEG purified, and stabilized with PEG-polylysine, containing 3 FRET pairs of Atto 488 and Atto 647N. The hTNFa-functionalized long rod was analyzed by agarose gel electrophoresis, TEM, and atomic force microscopy (AFM) in order to assess its quality, and were endotoxin-tested (Fig. 3a–c, Supplementary Fig. S4). Next we confirmed the coating of the long rod with the TNFa aptamer by incubating this nanostructure with a reverse complement sequence of that aptamer for 1 h at room temperature (Fig. 3d, Supplementary Fig. S5). To verify a specific binding of hTNFa protein to the long rod-TNFa aptamer rather than the non-functionalized long rod, (designated as long rod-no aptamer), we incubated each of these versions with recombinant hTNFa protein for 1 h followed by FACS binding assay. FACS analysis indicated the specific binding of the hTNFa protein to the long rod-TNFa aptamer-coated version (Fig. 3e). Also, as the TNFa aptamer used to coat the long rod nanostructure includes a short polyT linker of two T bases for improved accessibility, we performed the binding assay to assess its binding to TNFa protein in comparison to the original TNFa aptamer which does not include this linker. As can be seen, the addition of two T nucleotides did not affect TNFa aptamer binding to the TNFa protein (Supplementary Fig. S6).

Next, we examined the stability of the PEG-polylysine-coated long rod-TNFa aptamer in human serum at 37 °C over 10 days, by measuring total fluorescence efficiency, which indicated structure integrity. After 10 days, 34.7% of this aptamer-attached DNA nanostructures were degraded, indicating that the half-life of long rod-TNFa aptamer is longer than 10 days. Moreover, addition of DNase I to the human serum at 8th day resulted in immediate degradation (within 10 min) of more than 90% of the treated long rod-TNFa aptamer structures, as opposed to the untreated group, where 66.3% of the structures remained stable at the same time point (Fig. 3f).

**Figure 4:**
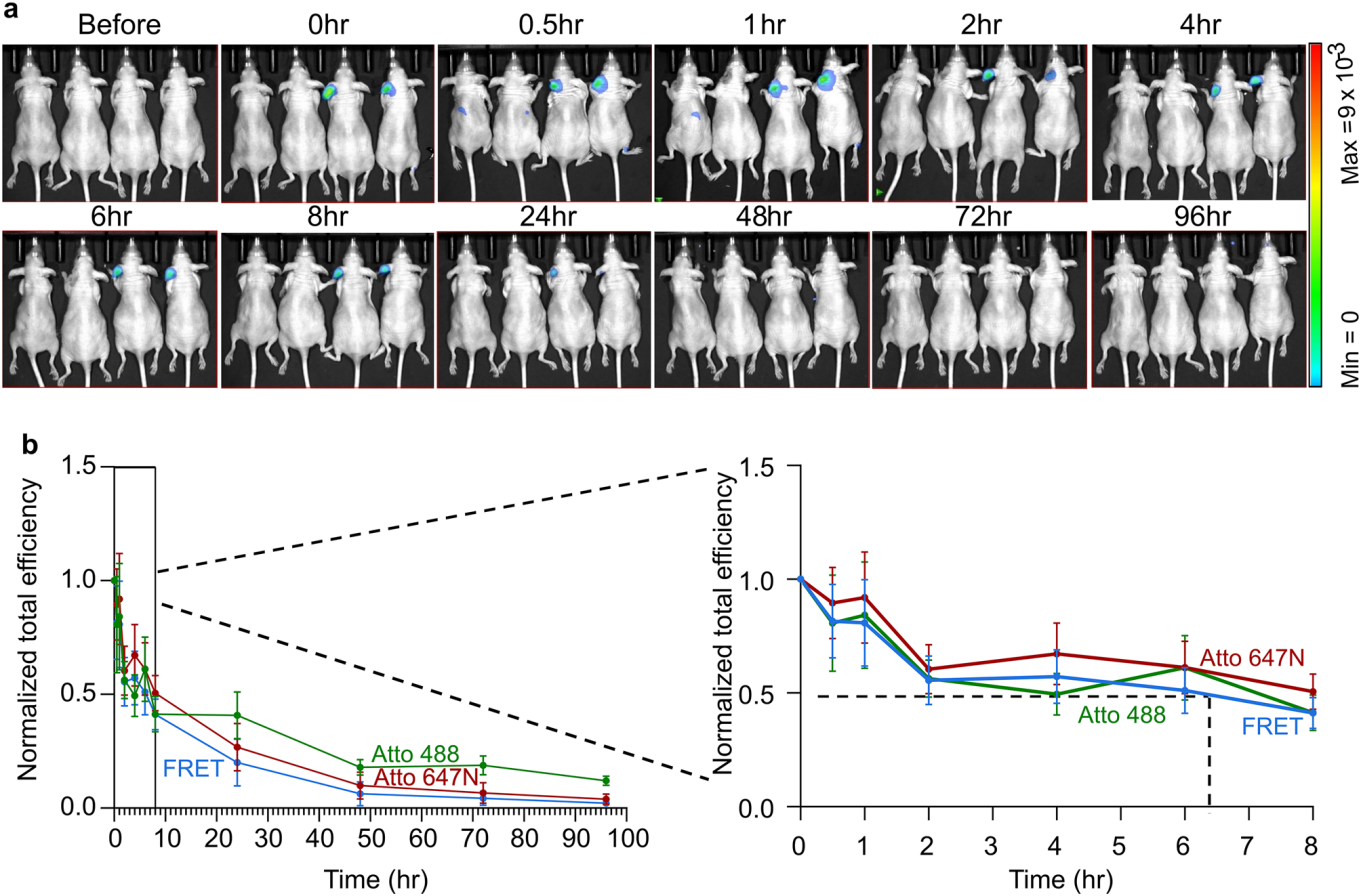
Biodistribution of long rod DNA origami following ear injection. (**a**) Live image analysis of total body biodistribution over time of the long rod DNA origami nanostructure after its injection into the left ear of nude mice. First two mice (from left to right) in each image were injected only with the vehicle. Heat map false color correlates to FRET levels. (**b**) Quantification of total efficiency fluorescence obtained in the left ear from (**a**). Same region of interest (ROI) was chosen around the injection area for each mouse and the fluorescent total efficiency of Atto 488, Atto 647N and FRET channels was measured in each ROI along time points. Calculations were performed as described in “Methods”. Marked rectangle is enlarged at the right. Data presented are the mean values ± SEM. Dashed lines in the magnified graph on the right indicate t_1/2_.

**Figure 5:**
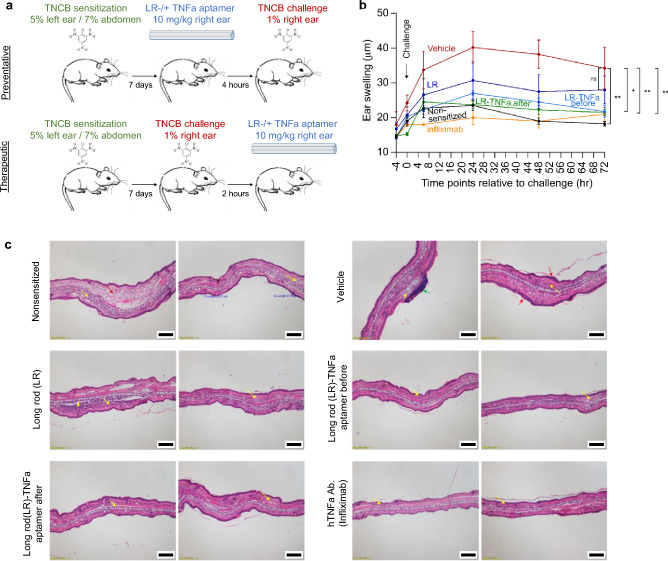
Long rod DNA origami coated with TNFa aptamers ameliorates inflammation indices in a DTHR mouse model. (**a**) Schematic illustration of TNCB-induced DTHR in humanized TNFa mice with two different treatment regimens, preventative (i.e., treatment is administered before the challenge with the hapten) and therapeutic (i.e., treatment is administered following the challenge) as indicated. (**b**) Ear thickness measurements before and at the indicated time points after TNCB challenge of nonsensitized mice (healthy), untreated TNCB-sensitized mice (vehicle), long rod-treated TNCB-sensitized mice, or infliximab-treated TNCB-sensitized mice. Both vehicle and healthy mouse groups were injected in their right ear with the buffer in which the long rod was dissolved in. All treatments were administered in mice sensitized with TNCB and challenged with TNCB, either before or after the challenge, as indicated. Long rod − / + TNFa aptamer was injected into the mouse right ear; infliximab was injected intravenously.Sensitization and challenge were performed as indicated in the method section. Data presented are the mean values ± SEM from two independent experiments, n = 4. Significance was determined by ANOVA with Tukey's multiple comparison test, and is indicated by asterisks (*P < 0.03; **P < 0.008; *ns* not significant). (**c**) Representative histopathological images of ear sections from the indicated mouse groups. All ear sections were fixed 72 h after ear challenge followed by hematoxylin and eosin staining. Red arrows indicate an increase of the dermis (healthy group), or the epidermis (vehicle group); yellow arrows indicate inflammatory infiltration; green arrows indicate crust formation. All scale bars are 200 µm.

In order to establish the PEG-polylysine-coated long rod potential to serve as a drug capable to elicit therapeutic effects in a spatially-confined manner, we utilized the DTHR experimental model in response to epicutaneous application of the hapten 2, 4, 6 trinitrochlorobenzene (TNCB). This reaction consists of two phases. The first phase is the induction or sensitization phase, during which Langherhans’ cells migrate from the sensitized area of the skin to draining lymph nodes and present haptene: major histocompatibility complex (MHC) complexes to T lymphocytes, leading to their subsequent activation. In the second phase, which is the challenge phase, reaction occurs as a result of migrating haptene-specific T lymphocytes to the site of antigen deposition i.e., challenge area of the skin (usually the ear), which is different from that of the sensitization skin area. This results in the subsequent production of proinflammatory cytokines, which recruit a variety of bystander cells, particularly macrophages, to the antigen challenge site, where they give rise to lesions, tissue necrosis and edema through the production of TNFa^29^. According to this experimental model, inflammation occurs in the ear, therefore we first characterized the kinetics of the long rod DNA nanostructure within the mouse ear. Half-life of the PEG-polylysine-coated long rod following its injection into the inner face of the ear was ~ 6 h (Fig. 4). Next, we induced an inflammatory reaction in humanized TNFa mice sensitized by either 5% or 7% TNCB in either their left ear or their abdomen, respectively, and challenged by applying 1% TNCB to their right ear. Non-sensitized mice, challenged with the hapten served as a negative control, i.e., healthy group. Mice were treated with either the PEG-polylysine-coated long rod-no aptamer or the functionalized version comprising 20 hTNFa aptamers (long rod-TNFa aptamer). Treatments were injected into the mouse right ear 4 h before TNCB challenge or 2 h following the challenge (Fig. 5a). Mice treated with infliximab, a chimeric monoclonal antibody used as a registered drug to treat a number of inflammatory conditions, served as a standard of care group. The vehicle group was composed of mice that were sensitized, challenged but treated only with the buffer. As can be seen in Fig. 5b, TNCB challenge of humanized TNFa mice that were also sensitized with TNCB induced antigen-specific swelling that peaked 24 h following challenge into the ear. Ear swelling response was still evident at 48 h and slightly declined 72 h post-challenge (vehicle group). In comparison, TNCB challenge of non-sensitized humanized TNFa mice elicited diminished ear swelling (healthy group). Interestingly, we found that mice treated with long rod nanostructures comprising TNFa aptamers exhibited reduced ear swelling in response to TNCB, compared with mice treated with long rod-no aptamer (Fig. 5b). Of note, injection into the ear was comparable between the different treatments in terms of fluorescent signal detected in the injected ear (Supplementary Fig. S7a). In order to evaluate the ability of the long rod-TNFa aptamer nanostructures to block TNFa activity and reduce the inflammatory reaction in-vivo, we determined ear tissue damage in mice by histopathology. Ears of humanized TNFa mice were sectioned and stained with hematoxylin and eosin in order to evaluate histopathological lesions after different treatments. Inflammation was present within the edematous dermis as a result from cellular infiltration of inflammatory cells. The inflammation grade was mild in healthy mice and in long rod-TNFa aptamer before challenge, or infliximab-treated groups; moderate in long rod-no aptamer, or long rod-TNFa aptamer after challenge-treated groups; moderate to severe in vehicle-treated group (Fig. 5c). Population distributions of infiltrating inflammatory cells were mostly similar among the different treatments, with lymphocytes and macrophages being the predominant infiltrating leukocytes whereas mast cells and eosinophils were present in low percentages (Supplementary Fig. S7b). Minimal epidermal thickness was significantly lower in long rod-TNFa aptamer-treated mice compared with long rod-no aptamer-treated mice (Supplementary Fig. S7c).

## Discussion

In this study, we describe the in-vivo kinetic of three DNA origami nanostructures having different shapes and aspect ratios and stabilized by PEG-polylysine. After choosing the optimal candidate, we next functionalized long rod nanostructure by attaching 20 hTNFa aptamers capable of targeting hTNFa protein. Next, we demonstrated the therapeutic potential of such functionalized copolymer-DNA origami hybrid nanoscale agents in response to an irritant reaction caused by a hapten using a DTHR mouse model in humanized TNFa mice. The Long rod-TNFa aptamer nanostructures exhibited efficient neutralization of hTNFa and reduction in local inflammation in all of the examined physiological parameters, including ear swelling, inflammation score and epidermal thickness, in both preventative (i.e., before challenge) and therapeutic (i.e., following challenge) treatments in comparison with vehicle-treated mice. Importantly, the improvement in inflammation parameters and ear tissue morphology in post challenge long rod-TNFa aptamer mice were comparable or better than that of mice treated before the challenge just with hTNFa aptamer. Notably, these effects were obtained within physiologically relevant concentrations and time ranges.

DNA origami-based aptamer-targeting therapeutics, such as the long rod-TNFa described here, have great potential in addressing key issues of specificity, efficacy, functionality and controllability by virtue of their advantageous properties. First, DNA origami technique and design process enables full addressability with nanometer precision, and control over size, shape, and surface chemistry of the desired nanostructure. Second, oligonucleotide therapeutics can be accurately designed to target any protein via SELEX or any mRNA based on their sequence, as was demonstrated in Moderna and Pfizer COVID-19 RNA-based vaccines, without the need for complex modeling and isolation in contrast to small molecules and proteins. Third, functionalization of origami nanostructures using active oligonucleotides (such as aptamers, small interfering RNAs, DNAzymes, ribozymes etc.) or even proteins or small molecules is relatively straightforward due to the predictability and accuracy of Watson–Crick base pairing or simplicity of the required chemical reaction. Moreover, the modularity and programmability of these copolymer-DNA origami hybrid nanoscale agents enables them exerting control across the spatial dimension of drug action by chelating its target in a spatially-confined manner, allowing to reduce the doses of the administered drug and thereby decreasing the systemic toxicity and possible side effects. And last, DNA origami has recently shown to be safe and non toxic in-vivo^30^, while PEG is a widely used biocompatible FDA approved polymer.

Moreover, the manufacturing of oligonucleotides as well as DNA origami structures is chemically-defined, fast, scalable, precise, highly reproducible, low-cost and does not require complex biological environments. In fact, one can purchase his own benchtop DNA printer and fold nanostructures in a beaker on an electric stove in a few hours^31^. These unique properties of nucleic-acid based therapeutics enable rapid response to health emergencies, global pandemics, biological terror, etc. making them critically essential^32–34^, as opposed to protein targeting therapeutics like mAbs, where the production process is relatively expensive, labor intensive and time consuming^35–37^. Additionally, the mAbs used for therapy are usually selected after deliberate vaccination, according to their high affinity towards an arbitrarily-chosen epitope of a pathogen or cellular antigen and therefore the selection is clearly skewed^4^. Moreover, several functional limitations of therapeutic mAbs have been raised including impaired interactions with the immune system, inadequate pharmacokinetics and tissue accessibility^38–41^. Conversely, the pharmacokinetics parameters and in-vivo stability of DNA nanostructures can be programmed and controlled by incorporations of varying amounts of base modification, artificial nucleoside analogs, such as N1-methylpseudouridine used in Pfizer COVID-19 vaccine^42–44^, and protective polymers without affecting their function^45^ advantages of higher stability, biocompatibility, as well as the ability to regulate the duration of action under most conditions.

Combined, our findings highlight hybrid polymer-DNA nanostructures as significant therapeutic agents with potential novel modality in the field of controllable and personalized medicine with improved precision and functionality. Furthermore, coating these PEG-DNA origami agents with a “cocktail”of aptamers against different targets could be envisioned in the next step of these therapeutic agents.

## Methods

### Design, preparation and folding of the DNA origami structures

Cuboid, short rod, long rod and long rod-TNFa aptamer origami nanostructures were designed and synthesized by Tilibit Nanosystems GmbH, using P7560 ssDNA (7560 base long) as scaffold strand **(**sequence is provided in Supplementary Information S1). Each structure contains three Atto 488 (donor) and Atto 647N (acceptor) FRET pairs. Staple strands were fabricated from synthetic DNA by Tilibit nanosystems. Assembly was carried out at a 1:4 scaffold:staple ratio (scaffold concentration 50 nM) in a buffer containing 5 mM Tris base, 1 mM EDTA, 5 mM NaCl and 20 mM MgCl_2_. Origami structures were prepared using the following annealing ramp: 15 min at 65 °C, 60 °C to 45 °C at − 1 °C/1 h in a thermoshaker MKR 13, Hettich Benelux. Subsequently, the DNA origami nanostructures were PEG purified and coated with PEG-polylysine (PEG_5K_-K_10_) as reported previously by Ponnuswamy et al.^25^. Briefly, purified nanostructures were mixed with PEG-polylysine (PEG_5K_-K_10_) at a final concentration of 1:1500 with P:N ratio (phosphates in DNA:nitrogen in amines) of 1:1. Sample was then incubated at room temperature for 1 h, during which electrostatic interactions occurred between the negatively charged origami structure and positively charged lysine, resulting in coated nanostructures. PEG_5K_-K_10_ was dissolved in 12 mM MgCl_2_, 40 mM Tris, 20 mM Acetate, 1 mM EDTA. Polydispersity index from gel permeation chromatography is between 1.00 and 1.20, and the average molecular weight as provided by the company is 6600 Da. Endotoxins were avoided and removed. Samples were dissolved in 5 mM Tris, 5 mM NaCl and 5 mM MgCl_2_ buffer. hTNFa aptamers were designed with an adapter and loaded on 20 specific sites (such that the aptamer part protrudes outwards) on the long rod nanostructures during the folding process (sequence is provided in Supplementary Information S1).

### Gel electrophoresis

DNA origami nanostructures were run on 2% agarose (Cat# 1712359, Bio-Lab) in 0.5 × TBE buffer (Cat# 01-871-1A, Sartorius) supplemented with 6 mM MgCl_2_, (Cat# AM9530, Invitrogen) and stained with 0.3 µg/ml ethidium bromide (Cat# 15585-011, Invitrogen) for 1 h at 80 V at room temperature. Running buffer contained 0.5X TBE and 6 mM MgCl_2_. 1 Kb DNA ladder (Cat# N3232S, New England Biolabs) was used as standard. Gel imaging was performed on either a Sapphire Biomolecular Imager RGB laser scanner, Azure Biosystems, or using Amersham ImageQuant™ 800 imaging system, Cytiva.

### Endotoxin detection test

A ToxinSensorTM Chromogenic LAL Endotoxin Assay Kit (Cat# L00350, GenScript) was used according to the manufacturer protocol to determine the endotoxin concentration in the DNA origami samples.

### Atomic force microscopy (AFM) imaging

All DNA nanostructures were imaged using Bruker (JPK) NanoWizard Ultra AFM III instrument in order to verify correct folding. Each sample (5 μl, concentration of 10–20 nM in 1 × TAE supplemented with 20 mM MgCl_2_) was deposited on a freshly cleaved mica (Grade V1, 10 mm, #50, TED PELLA, INC.) and left to absorb for 5 min. following a gentle wash using the same buffer. Finally, 1 ml of 1 × TAE, 20 mM MgCl_2_ buffer was added to the liquid cell (a small plastic ring that surrounds the mica). In TNFa binding experiments, long rod or long rod- TNFa aptamer were incubated with recombinant human TNFa protein for 2 or 5 h at 37 °C in a 1:25 ratio (nanostructure:TNFa protein).

All scans were performed in buffer at room temperature in FastScan-AC mode using ultra-short cantilevers with force constant of 0.3 N/m (Cat# 79427F7L1218, Nano World) at a scan rate of 10 Hz. Images were processed using JPK Data Processing v6.1.96, except for distances between TNFa aptamers over individual DNA origami structures that were analyzed manually.

### Transmission electron microscopy (TEM) imaging

400-mesh copper grids with continuous carbon film (Electron Microscopy Sciences) were freshly glow-discharged for 45 s at 35 mV (EMS K100X, Electron Microscopy Sciences) before incubation with samples. Samples (5 µl, 5 nM) were then applied onto the glow-discharged carbon-coated copper grids, incubated for 30 s at room temperature and stained with freshly prepared 2% (w/v) uranyl formate for negative staining of the DNA structures. TEM images were acquired on a Philips CM100 transmission electron microscope operated at 100 kV accelerating voltage using a single-tilt specimen holder. Images were collected with a AMT 4 Megapixel CCD camera.

### TNFa Reverse complement oligonucleotide and long rod incubation

Long rod or long rod-TNFa aptamer were incubated with a reverse complement oligonucleotide for TNFa aptamer tagged with Cy3 at either its 5ʹ or 3ʹ end for 1 h at room temperature in a 1:20 ratio (nanostructure:TNFa rev. comp. oligo, mole number). Following incubation, samples were run in gel electrophoresis as described above. Fluorescence of band intensities were measured under the following setting: Ex. 660 nm, Em. 715BP30 for Atto 647N detection; Ex. 535 nm, Em. 605BP40 for Cy3 detection.

### Binding assay

Binding of hTNFa protein to the long rod nanostructure was determined by Accuri C6 Plus Flow cytometer (Becton, Dickinson Biosciences) using Pierce streptavidin (SA) magnetic beads (Cat# TS-88817, Thermo scientific) and biotinylated recombinant human TNFa protein (Cat# BT210, R&D systems). SA magnetic beads (0.15 mg) were prewashed in PBS^-/-^ buffer containing 0.005% Tween-20, followed by incubation with biotinylated TNFa protein (3 ug) for 1 h at room temperature. Biotinylated TNFa protein-coated SA magnetic beads were then washed twice and incubated with either long rod-no aptamer, or long rod-TNFa aptamer in a 1:1 ratio (TNFa protein: nanostructure, mole number) for 1 h at 37 °C in PBS^−/−^ buffer containing 0.005% Tween-20. Samples were then washed twice followed by FACS readings. Fluorescence intensity was measured under the following setting: Ex. 640 nm, Em. 675/25 for Atto 647N detection. Analysis was performed using kaluza software version 2.1.

### Stability of long rod-TNFa aptamer in human serum in-vitro

Stability of long rod coated with hTNFa aptamer (300 nM) was examined in human serum (Cat# H4522, Sigma) at 37 °C. Samples containing a buffer, (in which the long rod-TNFa aptamer was dissolved in), in human serum served as control. Sample fluorescence was measured in different wavelengths (Ex. 500, Em. 570/20 for Atto 488 detection; Ex. 605, Em. 670/20 for Atto 647N detection; Ex. 500, Em. 670/20 for FRET detection) at the following time points: 0, 4, 24, 48, 96, 168, 192, 192.1, 192.5, 195, 216, 240 h using Ami HT spectral instruments imaging. After 8 days, half of the samples were treated with 40 units of DNase I (Cat# M0303S, Biolabs). Total fluorescence efficiency was normalized to time point 0 following subtraction of background signal obtained from control samples at each time point. Quantification was performed using Aura image analysis software (spectral instruments imaging, version 4.0.0).

Structure integrity was calculated by the following formula:

$$Structure\, integrity \left(\%\right)= \left(\frac{100}{AVG(\frac{1}{Fl\left(0\right)} \times 100 - AVG(FlDNase(240))} \right)\times \left(\frac{1}{Fl\left(t\right)} \times 100\right)-AVG(FlDNase(240))$$

$AVG$ = average; $Fl(t)$—fluorescent total efficiency at specific time point of each sample; $FlDNase(t)$—fluorescent total efficiency at specific time point of DNase-treated sample.

### Mice

Animal study was performed under the approval of the national council for experiments on animal subjects, Israel (application No. NPC-Sc-IL-2202-112-4). The research was conducted in accordance with ARRIVE guidelines. Animals in the study were housed, cared for and used in accordance with the international standards and the ILAR guide (the Institute for Laboratory Animal Research, Guide for the Care and Use of Laboratory Animals). C57BL/6 and nude mice were obtained from ENVIGO, Israel; humanized TNFa mice were obtained from genOway, France. Eight- to twelve- week-old mice were used in each experiment.

### In-vivo kinetics of DNA nanostructures

C57BL/6 mice were treated with cuboid, short rod or long rod DNA nanostructures either subcutaneously (200 µg), intraperitoneally (200 µg) or into the knee joints (100 µg). Mice treated with a vehicle (samples’ dissolving buffer containing 5 mM Tris pH 7, 1 mM EDTA pH 8, 5 mM NaCl_2_ and 5 mM MgCl_2_) served as control. Total body fluorescence was measured in different wavelengths (Ex. 500, Em. 570/20 for Atto 488 detection; Ex. 605, Em. 670/20 for Atto 647N detection; Ex. 500, Em. 670/20 for FRET detection) at the following time points: subcutaneous—0, 0.5, 1, 1.5, 2, 3.5, 5, 7, 11, 24, 48, 72 h; intraperitoneal—0, 0.4, 0.75, 1.3, 1.8, 2.6, 4, 5.75, 8.6 h; knee joints—0, 0.5, 1, 3, 5, 7, 24, 48 h. At each of the indicated time points, mice were anesthetized using isoflurane and imaged using Ami HT spectral instruments imaging. Fluorescent total efficiency was normalized to time point 0 following subtraction of background signal obtained from control mice at each time point. Quantification was performed using Aura image analysis software (spectral instruments imaging, version 4.0.0). Diffusion was calculated by measuring the vertical diameter of the highest fluorescent area using ImageJ. In the experiment of intraperitoneal administration, organs were excised and imaged 9 h following treatment with the DNA origami nanostructures.

For long rod-TNFa aptamer kinetics within the mouse ear, 200 µg of sample was injected into the inner face of the ear of C57BL/6 or nude mice. Total body fluorescence was measured in the same wavelengths specified above at the following time points: 0, 0.5, 1, 2, 4, 6, 8, 24, 48, 72, 96 h. At each of the indicated time points, mice were anesthetized and imaged as described above. Fluorescent total efficiency was normalized to time point 0 following subtraction of background signal obtained from the ear that was not injected at each time point. Quantification was performed as detailed above.

### Delayed-type hypersensitivity reaction (DTHR) mouse model

Humanized TNFa mice were sensitized with either 5% or 7% TNCB (4:1 mixture of acetone/olive oil) on either their left ear or their abdomen, respectively. One week following sensitization, mice were challenged with 1% TNCB in olive oil on their right ear. Treatments were administered into the mouse right ear either 4 h before challenge, or 2 h following the challenge at a final concentration of 1940 nM. Infliximab treatment was administered 4 h before challenge by intravenous injection at 5 mg/kg according to its guideline. Nonsensitized mice, challenged with the hapten at the right ear and treated with vehicles, served as a negative control, i.e., healthy group. Ear swelling was assessed by measuring ear thickness using a micrometer (Oditest, Kroeplin) before and after treatments and TNCB challenge. In mice treated with the long rod DNA nanostructure, total body fluorescence was measured in the same wavelengths specified above at the following time points relative to challenge: − 4, − 2, 0, + 2, + 4, + 24, + 48, + 72 h. At each of the indicated time points, mice were anesthetized and imaged as described above. Total fluorescence efficiency was normalized to the first time point of each experimental group following subtraction of background signal obtained from the ear that was not treated at each time point. Quantification was performed as detailed above.

### Histology

Samples of ear tissue from mice were harvested and fixed in 4% formaldehyde for 48 h. Next, the tissues were trimmed, placed in embedding cassettes and processed for paraffin embedding. Paraffin sections were cut (4 µm thick), transferred on glass slides, and stained with hematoxylin and eosin. Slides were then subjected to histopathological evaluation by collecting images using Olympus microscope (BX60, serial NO. 7D04032) equipped with a microscope's Camera (Olympus DP73, serial NO. OH05504) at objective magnification of $\times$ 4. A semi-quantitative analysis of inflammation grade was performed by counting the number of inflammatory cells (lymphocytes, macrophages, neutrophils, eosinophils and mast cells) per microscopic field. Additional histopathological parameters were examined, including epidermal thickness, the presence of necrosis and the presence of edema.

### Supplementary Information

Supplementary Information.

## Data Availability

The datasets used and/or analysed during the current study available from the corresponding author on a reasonable request.
