# Supplementary material for: Biodistribution and function of coupled polymer-DNA origami nanostructures
Source: Sci Rep. 2023 Nov 10;13:19567. doi: 10.1038/s41598-023-46351-1 (PMC10638432; doi:10.1038/s41598-023-46351-1)
Supplement: Supplementary file 1 — Supplementary Information. [file 41598_2023_46351_MOESM1_ESM.docx]

**Biodistribution and Function of Coupled Polymer-DNA Origami Nanostructures**

**Noah Joseph^1,3^, Anastasia Shapiro^1,2,3,*^, Ella Gillis^1^, Shirin Barkey^1^, Almogit Abu-Horowitz^1^, Ido Bachelet^1,4^, Boaz Mizrahi^2,4^**

1. Augmanity Nano, Rehovot, Israel
2. Faculty of Biotechnology and Food Engineering, Technion, Haifa 32000, Israel
3. These authors contributed equally to this work
4. These authors contributed equally to this work

* Address for Correspondence: [anastasia@augmanitynano.com](mailto:anastasia@augmanitynano.com)

**Supplementary Figure 1**

**
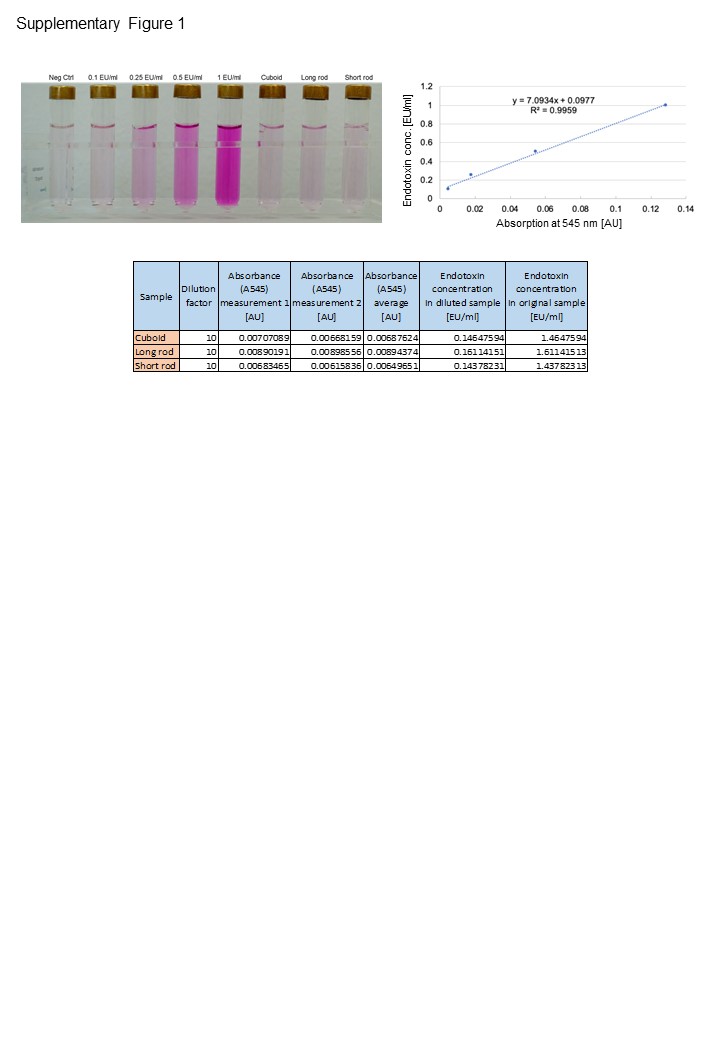
Figure S1. Endotoxin detection test of the DNA origami nanostructures.** Endotoxin assay of the indicated 10X diluted DNA origami nanostructures. Image shows the original test tubes of all the controls and the DNA origami samples. Graph provides the calibration curve for the endotoxin assay based on the indicated known endotoxin concentrations used as controls. Endotoxin concentrations in the DNA origami samples and their exact absorbance values measured at 545 nm are indicated in the presented table. Endotoxin concentrations were determined following the steps of PEG purification and PEG-polylysine addition.

**
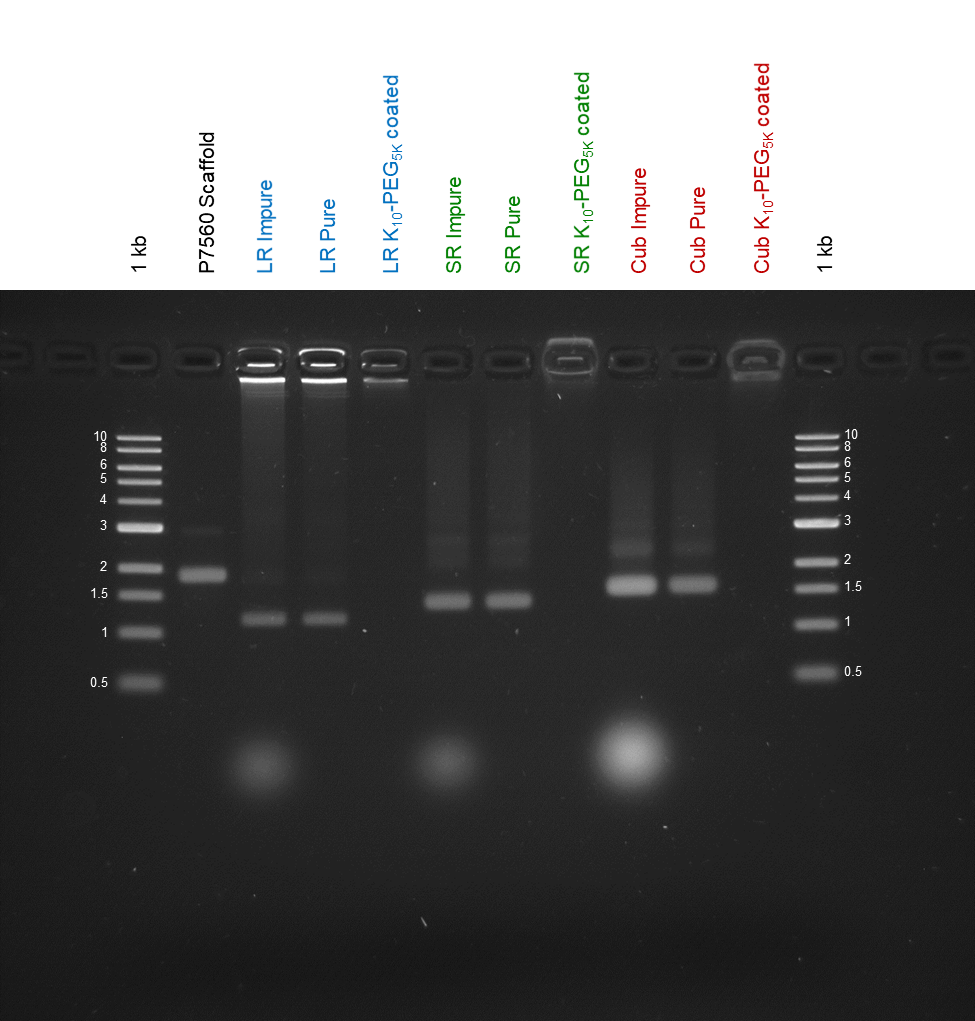
Supplementary Figure 2**

1 2 3 4 5 6 7 8 9 10 11 12

Lane #

**Figure S2. Full length gel electrophoresis of the DNA origami nanostructures.** Quality evaluation of the DNA origami nanostructures after assembly (lanes 3, 6, 9), after PEG purification (lanes 4, 7, 10) and after PEG-polylysine addition (lanes 5, 8, 11) as analyzed by gel electrophoresis. 1 kb double-stranded DNA was used as a ladder and bands are indicated on either side, numbers are in kb. Scaff. P7560 ssDNA scaffold; LR, long rod; SR, short rod; Cub, cuboid.

**Supplementary Figure 3**


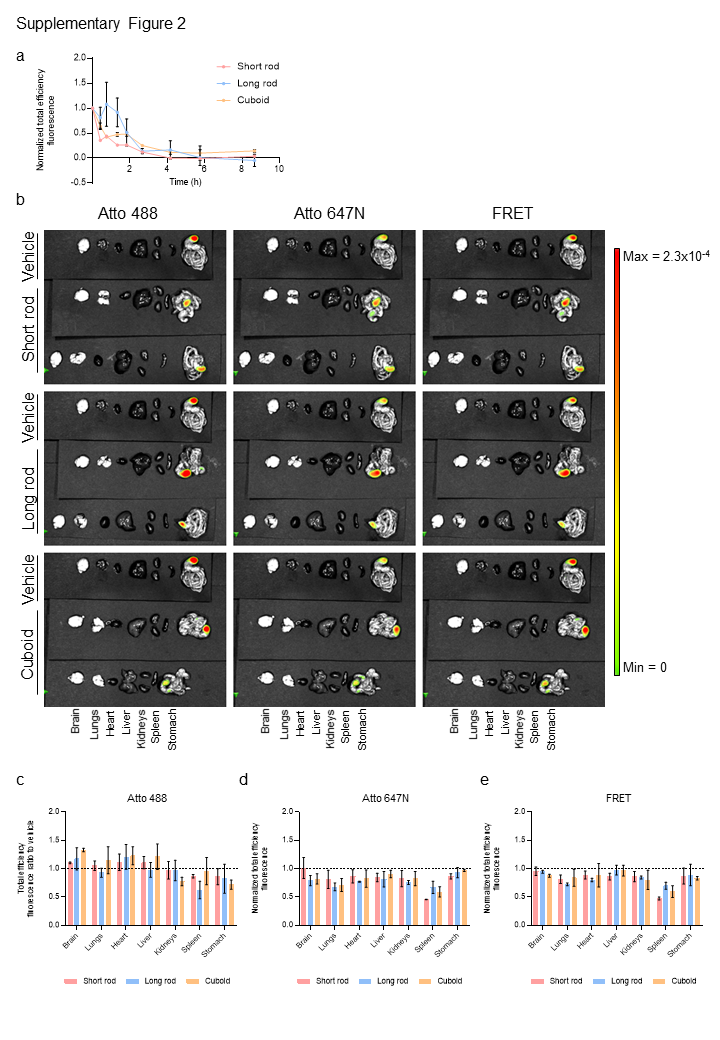


**Figure S3. Biodistribution of different DNA origami nanostructures following intraperitoneal injection. a.** Quantification of total efficiency fluorescence of the indicated DNA origami nanostructures obtained in live imaging of intraperitoneally-treated mice. Same region of interest (ROI) was chosen around the injection area for each mouse and the FRET fluorescent total efficiency of the indicated DNA origami nanostructures was measured in each ROI along time points. Calculations were performed as described in Methods. Data presented are the mean values ±SEM. **b.** *Ex-vivo* images of extracted organs from the intraperitoneally-treated mice. **c-e.** Quantification of total efficiency fluorescence obtained in organ images from b. The fluorescent total efficiency of Atto 488 (c), Atto 647N (d) and FRET (e) channels was measured for each indicated DNA origami nanostructure and the ratio relative to vehicle-treated mice was calculated. Data presented are the mean values ±SEM. Dashed lines indicate the vehicle normalized fluorescent level.

**Supplementary Figure 4**

**
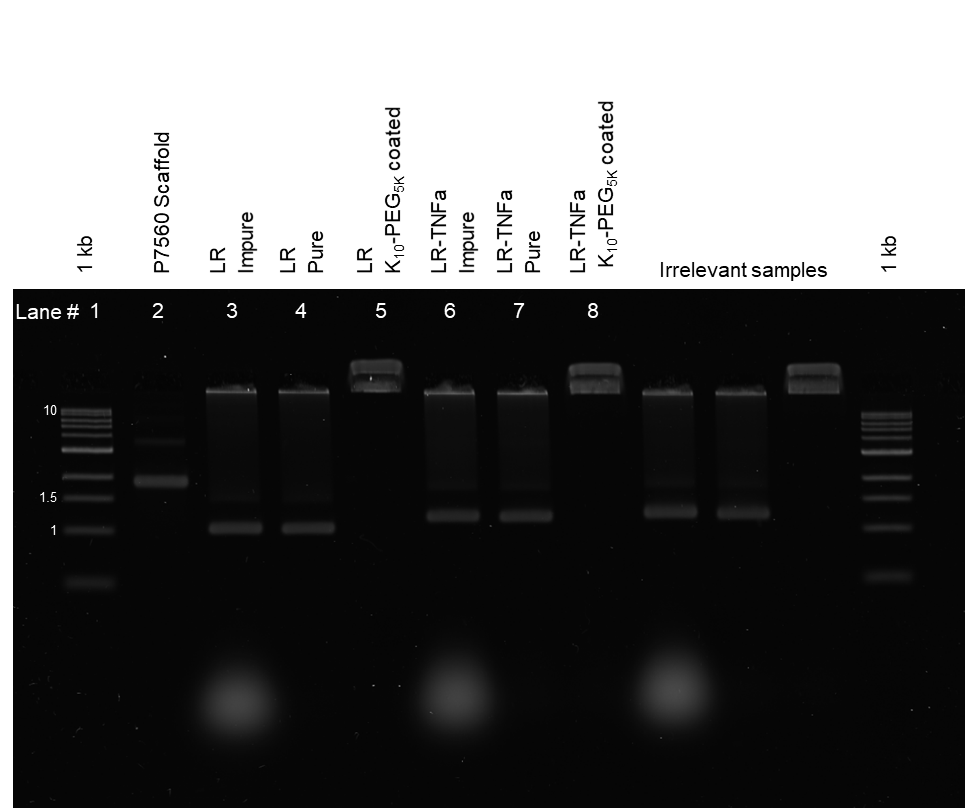
**a


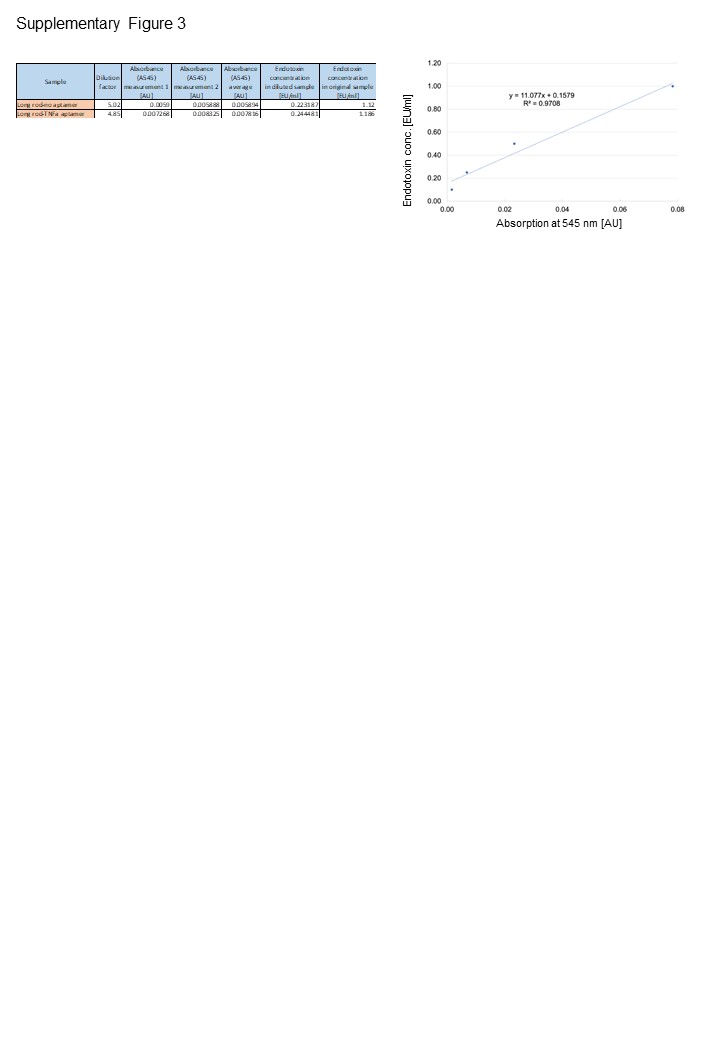
b

**Figure S4. Full length gel electrophoresis and endotoxin detection test of the long rod-TNFa aptamer.** **a.** Quality evaluation of either the long rod-no aptamer (LR) or the long rod-TNFa aptamer (LR-TNFa) after assembly (lanes 3, 6,), after PEG purification (lanes 4, 7) and after PEG-polylysine addition (lanes 5, 8) as analyzed by gel electrophoresis. 1 kb double-stranded DNA was used as a ladder and specific bands are indicated on the left side, numbers are in kb. Scaff., P7560 ssDNA scaffold. **b.** Endotoxin assay of TNFa-coated or uncoated long rod DNA origami nanostructure. The table presents the exact absorbance values measured at 545 nm for each DNA origami sample and the calculated endotoxin concentration using the calibration curve provided in the graph. Endotoxin concentrations were determined following the steps of PEG purification and PEG-polylysine addition.

**Supplementary Figure 5**

LR TNFa

LR

LR TNFa

LR

5’/3’ Cy3 Rev. Comp. - 5’ - 5’ - 3’ - 3’


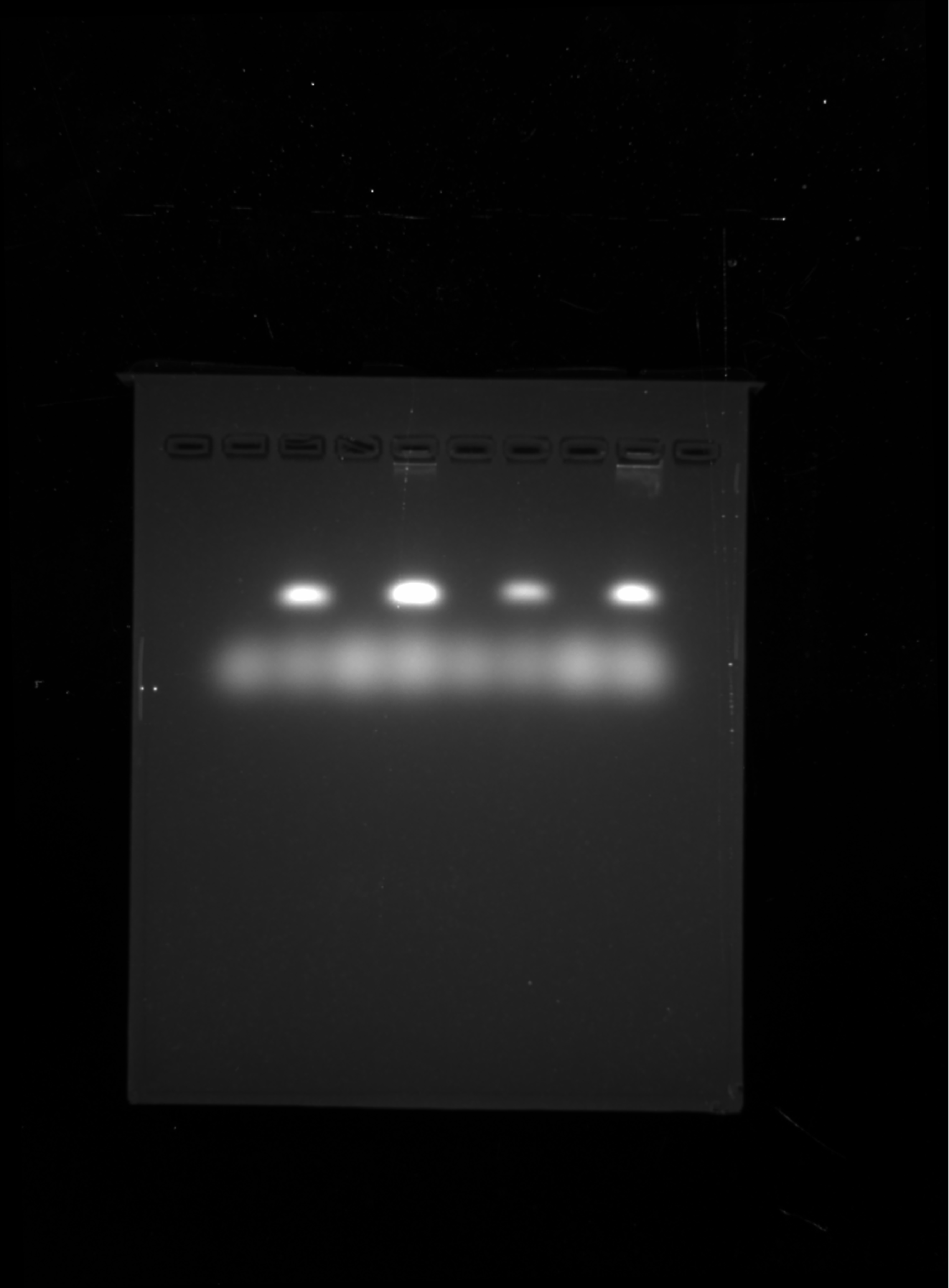


Cy3


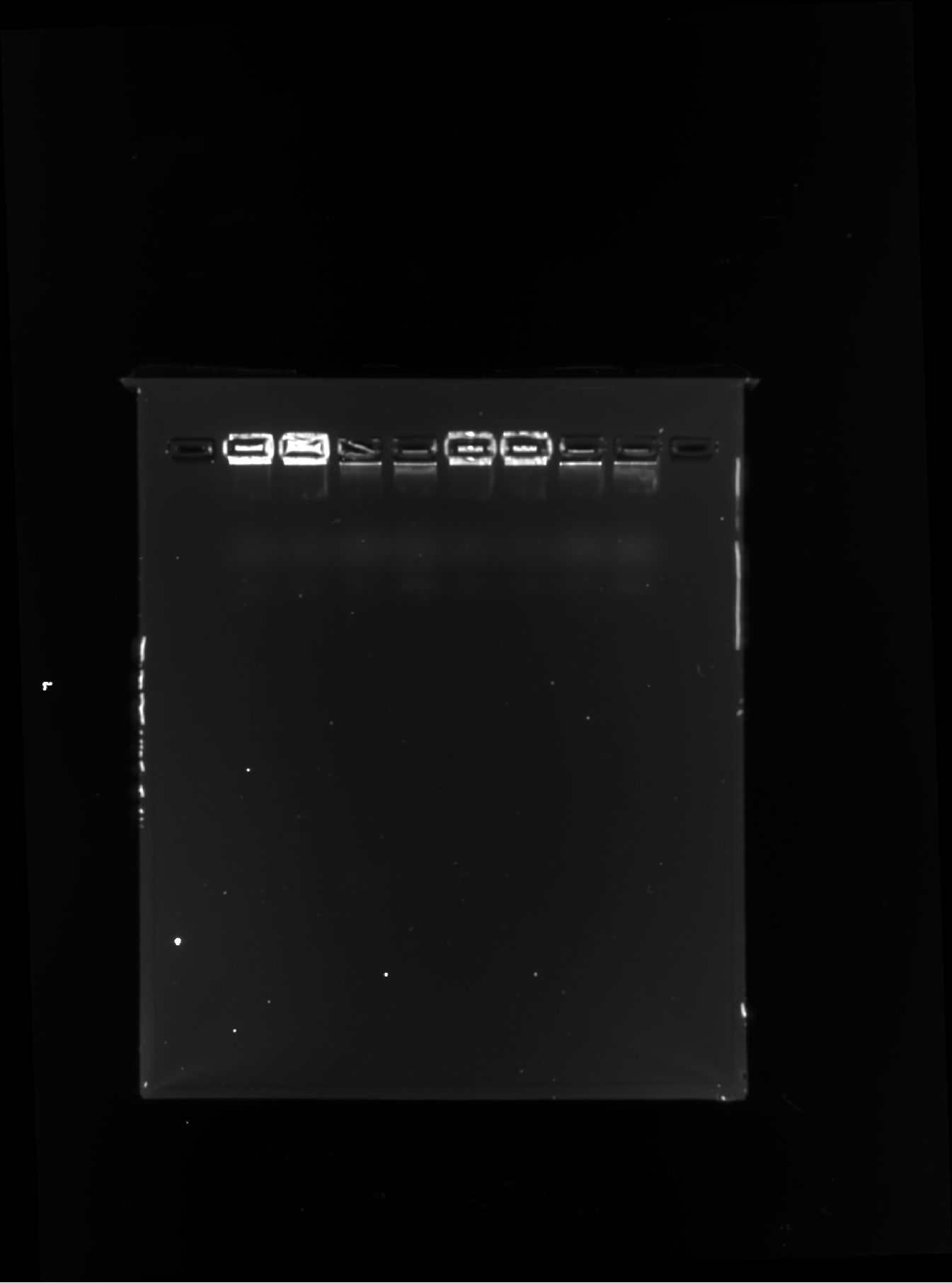


Atto647N

**Figure S5. Full length gel electrophoresis of a reverse complement sequence for the TNFa aptamer.** Incubation of a reverse complement sequence for the TNFa aptamer with either the long rod-no aptamer (LR) or the long rod-TNFa aptamer (LR TNFa) followed by gel electrophoresis. 5’ or 3’ indicates the end at which the reverse complement oligonucleotide was tagged with Cy3.

**Supplementary Figure 6**

TNFa ctrl aptamer

TNFa aptamer polyT linker

TNFa aptamer

SA beads

**Figure S6. Testing whether the addition of a short polyT linker has any effect on the binding of TNFa aptamer to TNFa protein.** FACS analysis of the binding between different versions of TNFa aptamer and TNFa protein-coated streptavidin (SA) beads.

**Supplementary Figure 7**


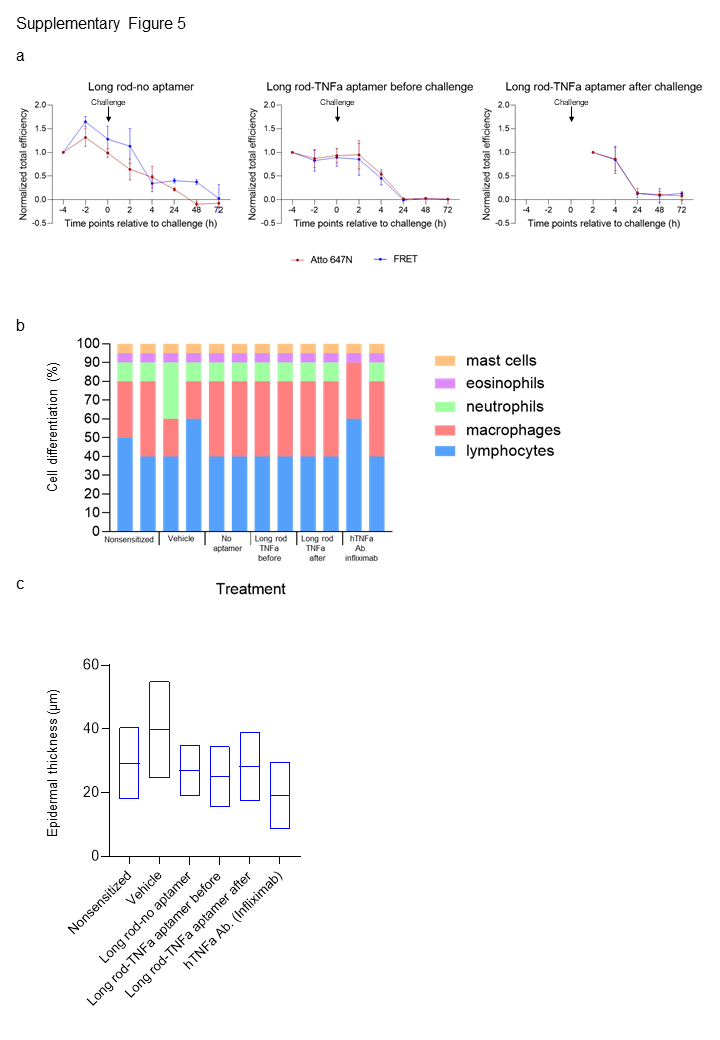


**Figure S7. Long rod-TNFa aptamer effect on histopathological parameters. a.** Quantification of total efficiency fluorescence obtained from live image analysis of total body biodistribution over time of the long rod DNA origami nanostructure after its injection into the right ear of humanized TNFa mice. Injection was either 4 hours before the challenge, or 2 hours following the challenge as indicated. Same ROI was chosen around the injection area for each mouse and the fluorescent total efficiency of Atto 647N and FRET channels was measured in each ROI along time points. Calculations were performed as described in Methods. Data presented are the mean values ±SEM. **b.** Cell type differentiation within infiltrated inflammatory cells as was counted in hematoxylin and eosin-stained ear sections of the indicated mouse groups. **c.** Averaged epidermal thickness range as was measured in hematoxylin and eosin-stained ear sections of the indicated mouse groups. Lines within floating bars indicate the mean.

**Supplementary Table 1**

|  | **No. of bases** | **Bare [g/mol]** | **PEG-oligolysin coated [g/mol]** |
| --- | --- | --- | --- |
| **Cuboid** | 15,474 | 5,059,998 | 15,272,838 |
| **Short rod** | 15,360 | 5,022,720 | 15,160,320 |
| **Long rod** | 15,240 | 4,983,480 | 15,041,880 |

**Supplementary Table 1:** The molecular weight of the indicated nanostructures before and after their coating with PEG-polylysine (PEG_5K_-K_10_).

**Supplementary Table 2**

| **#** | **Aptamer position / nm** |
| --- | --- |
| 1 | 0 (end) |
| 2 | 0 (end) |
| 3 | 20.74 |
| 4 | 28.22 |
| 5 | 46.24 |
| 6 | 49.64 |
| 7 | 63.58 |
| 8 | 67.32 |
| 9 | 81.94 |
| 10 | 85 |
| 11 | 92.82 |
| 12 | 113.9 |
| 13 | 131.92 |
| 14 | 142.46 |
| 15 | 149.26 |
| 16 | 159.8 |
| 17 | 178.16 |
| 18 | 189.04 |
| 19 | 214.54 (end) |
| 20 | 214.54 (end) |

**Supplementary Table 2:** TNFa aptamer positions/locations on long rod-TNFa nanostructure.

**Supplementary Data**

**P7560 ssDNA Scaffold**

**> P7560 [length=7560] [topology=circular], complete sequence.**

AGCTTGGCACTGGCCGTCGTTTTACAACGTCGTGACTGGGAAAACCCTGGCGTTACCCAACTTAATCGCCTTGCAGCACATCCCCCTTTCGCCAGCTGGCGTAATAGCGAAGAGGCCCGCACCGATCGCCCTTCCCAACAGTTGCGCAGCCTGAATGGCGAATGGCGCTTTGCCTGGTTTCCGGCACCAGAAGCGGTGCCGGAAAGCTGGCTGGAGTGCGATCTTCCTGAGGCCGATACTGTCGTCGTCCCCTCAAACTGGCAGATGCACGGTTACGATGCGCCCATCTACACCAACGTGACCTATCCCATTACGGTCAATCCGCCGTTTGTTCCCACGGAGAATCCGACGGGTTGTTACTCGCTCACATTTAATGTTGATGAAAGCTGGCTACAGGAAGGCCAGACGCGAATTATTTTTGATGGCGTTCCTATTGGTTAAAAAATGAGCTGATTTAACAAAAATTTAATGCGAATTTTAACAAAATATTAACGTTTACAATTTAAATATTTGCTTATACAATCTTCCTGTTTTTGGGGCTTTTCTGATTATCAACCGGGGTACATATGATTGACATGCTAGTTTTACGATTACCGTTCATCGATTCTCTTGTTTGCTCCAGACTCTCAGGCAATGACCTGATAGCCTTTGTAGATCTCTCAAAAATAGCTACCCTCTCCGGCATTAATTTATCAGCTAGAACGGTTGAATATCATATTGATGGTGATTTGACTGTCTCCGGCCTTTCTCACCCTTTTGAATCTTTACCTACACATTACTCAGGCATTGCATTTAAAATATATGAGGGTTCTAAAAATTTTTATCCTTGCGTTGAAATAAAGGCTTCTCCCGCAAAAGTATTACAGGGTCATAATGTTTTTGGTACAACCGATTTAGCTTTATGCTCTGAGGCTTTATTGCTTAATTTTGCTAATTCTTTGCCTTGCCTGTATGATTTATTGGATGTTAATGCTACTACTATTAGTAGAATTGATGCCACCTTTTCAGCTCGCGCCCCAAATGAAAATATAGCTAAACAGGTTATTGACCATTTGCGAAATGTATCTAATGGTCAAACTAAATCTACTCGTTCGCAGAATTGGGAATCAACTGTTATATGGAATGAAACTTCCAGACACCGTACTTTAGTTGCATATTTAAAACATGTTGAGCTACAGCATTATATTCAGCAATTAAGCTCTAAGCCATCCGCAAAAATGACCTCTTATCAAAAGGAGCAATTAAAGGTACTCTCTAATCCTGACCTGTTGGAGTTTGCTTCCGGTCTGGTTCGCTTTGAAGCTCGAATTAAAACGCGATATTTGAAGTCTTTCGGGCTTCCTCTTAATCTTTTTGATGCAATCCGCTTTGCTTCTGACTATAATAGTCAGGGTAAAGACCTGATTTTTGATTTATGGTCATTCTCGTTTTCTGAACTGTTTAAAGCATTTGAGGGGGATTCAATGAATATTTATGACGATTCCGCAGTATTGGACGCTATCCAGTCTAAACATTTTACTATTACCCCCTCTGGCAAAACTTCTTTTGCAAAAGCCTCTCGCTATTTTGGTTTTTATCGTCGTCTGGTAAACGAGGGTTATGATAGTGTTGCTCTTACTATGCCTCGTAATTCCTTTTGGCGTTATGTATCTGCATTAGTTGAATGTGGTATTCCTAAATCTCAACTGATGAATCTTTCTACCTGTAATAATGTTGTTCCGTTAGTTCGTTTTATTAACGTAGATTTTTCTTCCCAACGTCCTGACTGGTATAATGAGCCAGTTCTTAAAATCGCATAAGGTAATTCACAATGATTAAAGTTGAAATTAAACCATCTCAAGCCCAATTTACTACTCGTTCTGGTGTTTCTCGTCAGGGCAAGCCTTATTCACTGAATGAGCAGCTTTGTTACGTTGATTTGGGTAATGAATATCCGGTTCTTGTCAAGATTACTCTTGATGAAGGTCAGCCAGCCTATGCGCCTGGTCTGTACACCGTTCATCTGTCCTCTTTCAAAGTTGGTCAGTTCGGTTCCCTTATGATTGACCGTCTGCGCCTCGTTCCGGCTAAGTAACATGGAGCAGGTCGCGGATTTCGACACAATTTATCAGGCGATGATACAAATCTCCGTTGTACTTTGTTTCGCGCTTGGTATAATCGCTGGGGGTCAAAGATGAGTGTTTTAGTGTATTCTTTTGCCTCTTTCGTTTTAGGTTGGTGCCTTCGTAGTGGCATTACGTATTTTACCCGTTTAATGGAAACTTCCTCATGAAAAAGTCTTTAGTCCTCAAAGCCTCTGTAGCCGTTGCTACCCTCGTTCCGATGCTGTCTTTCGCTGCTGAGGGTGACGATCCCGCAAAAGCGGCCTTTAACTCCCTGCAAGCCTCAGCGACCGAATATATCGGTTATGCGTGGGCGATGGTTGTTGTCATTGTCGGCGCAACTATCGGTATCAAGCTGTTTAAGAAATTCACCTCGAAAGCAAGCTGATAAACCGATACAATTAAAGGCTCCTTTTGGAGCCTTTTTTTTGGAGATTTTCAACGTGAAAAAATTATTATTCGCAATTCCTTTAGTTGTTCCTTTCTATTCTCACTCCGCTGAAACTGTTGAAAGTTGTTTAGCAAAATCCCATACAGAAAATTCATTTACTAACGTCTGGAAAGACGACAAAACTTTAGATCGTTACGCTAACTATGAGGGCTGTCTGTGGAATGCTACAGGCGTTGTAGTTTGTACTGGTGACGAAACTCAGTGTTACGGTACATGGGTTCCTATTGGGCTTGCTATCCCTGAAAATGAGGGTGGTGGCTCTGAGGGTGGCGGTTCTGAGGGTGGCGGTTCTGAGGGTGGCGGTACTAAACCTCCTGAGTACGGTGATACACCTATTCCGGGCTATACTTATATCAACCCTCTCGACGGCACTTATCCGCCTGGTACTGAGCAAAACCCCGCTAATCCTAATCCTTCTCTTGAGGAGTCTCAGCCTCTTAATACTTTCATGTTTCAGAATAATAGGTTCCGAAATAGGCAGGGGGCATTAACTGTTTATACGGGCACTGTTACTCAAGGCACTGACCCCGTTAAAACTTATTACCAGTACACTCCTGTATCATCAAAAGCCATGTATGACGCTTACTGGAACGGTAAATTCAGAGACTGCGCTTTCCATTCTGGCTTTAATGAGGATTTATTTGTTTGTGAATATCAAGGCCAATCGTCTGACCTGCCTCAACCTCCTGTCAATGCTGGCGGCGGCTCTGGTGGTGGTTCTGGTGGCGGCTCTGAGGGTGGTGGCTCTGAGGGTGGCGGTTCTGAGGGTGGCGGCTCTGAGGGAGGCGGTTCCGGTGGTGGCTCTGGTTCCGGTGATTTTGATTATGAAAAGATGGCAAACGCTAATAAGGGGGCTATGACCGAAAATGCCGATGAAAACGCGCTACAGTCTGACGCTAAAGGCAAACTTGATTCTGTCGCTACTGATTACGGTGCTGCTATCGATGGTTTCATTGGTGACGTTTCCGGCCTTGCTAATGGTAATGGTGCTACTGGTGATTTTGCTGGCTCTAATTCCCAAATGGCTCAAGTCGGTGACGGTGATAATTCACCTTTAATGAATAATTTCCGTCAATATTTACCTTCCCTCCCTCAATCGGTTGAATGTCGCCCTTTTGTCTTTGGCGCTGGTAAACCATATGAATTTTCTATTGATTGTGACAAAATAAACTTATTCCGTGGTGTCTTTGCGTTTCTTTTATATGTTGCCACCTTTATGTATGTATTTTCTACGTTTGCTAACATACTGCGTAATAAGGAGTCTTAATCATGCCAGTTCTTTTGGGTATTCCGTTATTATTGCGTTTCCTCGGTTTCCTTCTGGTAACTTTGTTCGGCTATCTGCTTACTTTTCTTAAAAAGGGCTTCGGTAAGATAGCTATTGCTATTTCATTGTTTCTTGCTCTTATTATTGGGCTTAACTCAATTCTTGTGGGTTATCTCTCTGATATTAGCGCTCAATTACCCTCTGACTTTGTTCAGGGTGTTCAGTTAATTCTCCCGTCTAATGCGCTTCCCTGTTTTTATGTTATTCTCTCTGTAAAGGCTGCTATTTTCATTTTTGACGTTAAACAAAAAATCGTTTCTTATTTGGATTGGGATAAATAATATGGCTGTTTATTTTGTAACTGGCAAATTAGGCTCTGGAAAGACGCTCGTTAGCGTTGGTAAGATTCAGGATAAAATTGTAGCTGGGTGCAAAATAGCAACTAATCTTGATTTAAGGCTTCAAAACCTCCCGCAAGTCGGGAGGTTCGCTAAAACGCCTCGCGTTCTTAGAATACCGGATAAGCCTTCTATATCTGATTTGCTTGCTATTGGGCGCGGTAATGATTCCTACGATGAAAATAAAAACGGCTTGCTTGTTCTCGATGAGTGCGGTACTTGGTTTAATACCCGTTCTTGGAATGATAAGGAAAGACAGCCGATTATTGATTGGTTTCTACATGCTCGTAAATTAGGATGGGATATTATTTTTCTTGTTCAGGACTTATCTATTGTTGATAAACAGGCGCGTTCTGCATTAGCTGAACATGTTGTTTATTGTCGTCGTCTGGACAGAATTACTTTACCTTTTGTCGGTACTTTATATTCTCTTATTACTGGCTCGAAAATGCCTCTGCCTAAATTACATGTTGGCGTTGTTAAATATGGCGATTCTCAATTAAGCCCTACTGTTGAGCGTTGGCTTTATACTGGTAAGAATTTGTATAACGCATATGATACTAAACAGGCTTTTTCTAGTAATTATGATTCCGGTGTTTATTCTTATTTAACGCCTTATTTATCACACGGTCGGTATTTCAAACCATTAAATTTAGGTCAGAAGATGAAATTAACTAAAATATATTTGAAAAAGTTTTCTCGCGTTCTTTGTCTTGCGATTGGATTTGCATCAGCATTTACATATAGTTATATAACCCAACCTAAGCCGGAGGTTAAAAAGGTAGTCTCTCAGACCTATGATTTTGATAAATTCACTATTGACTCTTCTCAGCGTCTTAATCTAAGCTATCGCTATGTTTTCAAGGATTCTAAGGGAAAATTAATTAATAGCGACGATTTACAGAAGCAAGGTTATTCACTCACATATATTGATTTATGTACTGTTTCCATTAAAAAAGGTAATTCAAATGAAATTGTTAAATGTAATTAATTTTGTTTTCTTGATGTTTGTTTCATCATCTTCTTTTGCTCAGGTAATTGAAATGAATAATTCGCCTCTGCGCGATTTTGTAACTTGGTATTCAAAGCAATCAGGCGAATCCGTTATTGTTTCTCCCGATGTAAAAGGTACTGTTACTGTATATTCATCTGACGTTAAACCTGAAAATCTACGCAATTTCTTTATTTCTGTTTTACGTGCAAATAATTTTGATATGGTAGGTTCTAACCCTTCCATTATTCAGAAGTATAATCCAAACAATCAGGATTATATTGATGAATTGCCATCATCTGATAATCAGGAATATGATGATAATTCCGCTCCTTCTGGTGGTTTCTTTGTTCCGCAAAATGATAATGTTACTCAAACTTTTAAAATTAATAACGTTCGGGCAAAGGATTTAATACGAGTTGTCGAATTGTTTGTAAAGTCTAATACTTCTAAATCCTCAAATGTATTATCTATTGACGGCTCTAATCTATTAGTTGTTAGTGCTCCTAAAGATATTTTAGATAACCTTCCTCAATTCCTTTCAACTGTTGATTTGCCAACTGACCAGATATTGATTGAGGGTTTGATATTTGAGGTTCAGCAAGGTGATGCTTTAGATTTTTCATTTGCTGCTGGCTCTCAGCGTGGCACTGTTGCAGGCGGTGTTAATACTGACCGCCTCACCTCTGTTTTATCTTCTGCTGGTGGTTCGTTCGGTATTTTTAATGGCGATGTTTTAGGGCTATCAGTTCGCGCATTAAAGACTAATAGCCATTCAAAAATATTGTCTGTGCCACGTATTCTTACGCTTTCAGGTCAGAAGGGTTCTATCTCTGTTGGCCAGAATGTCCCTTTTATTACTGGTCGTGTGACTGGTGAATCTGCCAATGTAAATAATCCATTTCAGACGATTGAGCGTCAAAATGTAGGTATTTCCATGAGCGTTTTTCCTGTTGCAATGGCTGGCGGTAATATTGTTCTGGATATTACCAGCAAGGCCGATAGTTTGAGTTCTTCTACTCAGGCAAGTGATGTTATTACTAATCAAAGAAGTATTGCTACAACGGTTAATTTGCGTGATGGACAGACTCTTTTACTCGGTGGCCTCACTGATTATAAAAACACTTCTCAGGATTCTGGCGTACCGTTCCTGTCTAAAATCCCTTTAATCGGCCTCCTGTTTAGCTCCCGCTCTGATTCTAACGAGGAAAGCACGTTATACGTGCTCGTCAAAGCAACCATAGTACGCGCCCTGTAGCGGCGCATTAAGCGCGGCGGGTGTGGTGGTTACGCGCAGCGTGACCGCTACACTTGCCAGCGCCCTAGCGCCCGCTCCTTTCGCTTTCTTCCCTTCCTTTCTCGCCACGTTCGCCGGCTTTCCCCGTCAAGCTCTAAATCGGGGGCTCCCTTTAGGGTTCCGATTTAGTGCTTTACGGCACCTCGACCCCAAAAAACTTGATTTGGGTGATGGTTCACGTAGTGGGCCATCGCCCTGATAGACGGTTTTTCGCCCTTTGACGTTGGAGTCCACGTTCTTTAATAGTGGACTCTTGTTCCAAACTGGAACAACACTCAACCCTATCTCGGGCTATTCTTTTGATTTATAAGGGATTTTGCCGATTTCGGAACCACCATCAAACAGGATTTTCGCCTGCTGGGGCAAACCAGCGTGGACCGCTTGCTGCAACTCTCTCAGGGCCAGGCGGTGAAGGGCAATCAGCTGTTGCCCGTCTCACTGGTGAAAAGAAAAACCACCCTGGCGCCCAATACGCAAACCGCCTCTCCCCGCGCGTTGGCCGATTCATTAATGCAGCTGGCACGACAGGTTTCCCGACTGGAAAGCGGGCAGTGAGCGCAACGCAATTAATGTGAGTTAGCTCACTCATTAGGCACCCCAGGCTTTACACTTTATGCTTCCGGCTCGTATGTTGTGTGGAATTGTGAGCGGATAACAATTTCACACAGGAAACAGCTATGACCATGATTACGAATTCGAGCTCGGTACCCGGGGATCCTCCGTCTTTATCGAGGTAACAAGCACCACGTAGCTTAAGCCCTGTTTACTCATTACACCAACCAGGAGGTCAGAGTTCGGAGAAATGATTTATGTGAAATGCGTCAGCCGATTCAAGGCCCCTATATTCGTGCCCACCGACGAGTTGCTTACAGATGGCAGGGCCGCACTGTCGGTATCATAGAGTCACTCCAGGGCGAGCGTAAATAGATTAGAAGCGGGGTTATTTTGGCGGGACATTGTCATAAGGTTGACAATTCAGCACTAAGGACACTTAAGTCGTGCGCATGAATTCACAACCACTTAGAAGAACATCCACCCTGGCTTCTCCTGAGAA

**hTNFa ssDNA aptamer**

**> hTNFa [length=25], complete sequence.**

/5ATTO647NN/TGGTGGATGGCGCAGTCGGCGACAA

**hTNFa long rod ssDNA aptamer**

**> hTNFa long rod [length=27], complete sequence.**

/5ATTO647NN/TTTGGTGGATGGCGCAGTCGGCGACAA

**Control aptamer**

**> Control [length=25], complete sequence.**

/5ATTO647NN/TTTGGTGGATGGCGCAGTCGGCGACAA

**5Cy3 Rev. Comp. hTNFa ssDNA aptamer**

**> 5Cy3 Rev. Comp. hTNFa [length=25], complete sequence.**

/5Cy3/TTGTCGCCGACTGCGCCATCCACCA

**3Cy3 Rev. Comp. hTNFa ssDNA aptamer**

**> 3Cy3 Rev. Comp. hTNFa [length=25], complete sequence.**

TTGTCGCCGACTGCGCCATCCACCA/3Cy3Sp/
